# Neutrophil-lymphocyte ratio as a predictor of delirium in older internal medicine patients: a prospective cohort study

**DOI:** 10.1186/s12877-021-02284-w

**Published:** 2021-05-25

**Authors:** Yanli Zhao, Jirong Yue, Peng Lei, Taiping Lin, Xuchao Peng, Dongmei Xie, Langli Gao, Xiaoyu Shu, Chenkai Wu

**Affiliations:** 1 Department of Geriatrics and National Clinical Research Center for Geriatrics, West China Hospital, Sichuan University, 610041 Chengdu, Sichuan Province China; 2 Department of Neurology, State Key Laboratory of Biotherapy, West China Hospital, Sichuan University, 610041 Chengdu, Sichuan Province China; 3 Global Health Research Center, Duke Kunshan University, 215300 Kunshan, Jiangsu Province China

**Keywords:** Delirium, Older people, Neutrophil-lymphocyte ratio

## Abstract

**Backgrounds:**

Delirium is a common neuropsychiatric syndrome in older hospitalized patients. Previous studies have suggested that inflammation and oxidative stress contribute to the pathophysiology of delirium. However, it remains unclear whether neutrophil-lymphocyte ratio (NLR), an indicator of systematic inflammation, is associated with delirium. This study aimed to investigate the value of NLR as an independent risk factor for delirium among older hospitalized patients.

**Methods:**

We conducted a prospective study of 740 hospitalized patients aged ≥ 70 years in the geriatric ward of West China Hospital of Sichuan University. Neutrophil and lymphocyte counts were collected within 24 h after hospital admission. Delirium was assessed on admission and every 48 h thereafter. We used the receiver operating characteristic analysis to assess the ability of the NLR for predicting delirium. The optimal cut-point value of the NLR was determined based on the highest Youden index (sensitivity + specificity − 1). Patients were categorized according to the cut-point value and quartiles of NLR, respectively. We then used logistic regression to identify the unadjusted and adjusted associations between NLR as a categorical variable and delirium.

**Results:**

The optimal cut-point value of NLR for predicting delirium was 3.626 (sensitivity: 75.2 %; specificity: 63.4 %; Youden index: 0.386). The incidence of delirium was significantly higher in patients with NLR > 3.626 than NLR ≤ 3.626 (24.5 % vs. 5.8 %; *P* < 0.001). Significantly fewer patients in the first quartile of NLR experienced delirium than in the third (4.3 % vs. 20.0 %; *P* < 0.001) and fourth quartiles of NLR (4.3 % vs. 24.9 %; *P* < 0.001). Results from the multivariable logistic regression models showed that NLR was independently associated with delirium.

**Conclusions:**

NLR is a simple and practical marker that can predict the development of delirium in older internal medicine patients.

**Supplementary Information:**

The online version contains supplementary material available at 10.1186/s12877-021-02284-w.

## Background

Delirium is an acute neuropsychiatric syndrome characterized by disturbances in consciousness, cognition and attention [1]. Delirium is a common complication among hospitalized older patients and associated with a variety of adverse outcomes, including cognitive impairment, prolonged hospital stay, functional disability, and mortality [2–5].

Diagnosis of delirium is primarily based on clinical observation and its underlying pathophysiology is not entirely understood [6]. Inflammation and oxidative stress have been reported to play a key role in the development of delirium [7, 8]. Systemic inflammation could lead to neuro-inflammation and resultant delirium through activation of brain parenchymal cells and an expression of cytokines in the brain [9]. Recent studies have demonstrated associations between traditional inflammatory markers and delirium, such as C-reactive protein (CRP), interleukin (IL)-6, IL-8, IL-2, and tumor necrosis factor (TNF) [10–14]. However, the clinical utility of these biomarkers has been limited due to relatively high costs and inconvenience to measure. Hence, identification of simple inflammatory markers, easily available in every health care setting, is essential to improve delirium recognition and prediction among older patients.

The neutrophil-lymphocyte ratio (NLR), obtained easily from the circulation, is an indicator of inflammation and oxidative stress [15]. NLR has been applied to prognosis evaluation in various disciplines including malignancies, cardiovascular diseases, kidney diseases, and sepsis [16–20]. Additionally, several studies have reported a relationship between increased NLR and neurological or psychiatric conditions, such as Alzheimer’s disease, schizophrenia, Parkinson’s disease, ischemic stroke as well as memory disorders [15, 21, 22]. However, studies investigating the effect of NLR on delirium in older hospitalized patients are rare.

The present study aimed to explore the relationship between NLR and delirium, and to examine the value of NLR as a predictor of delirium in older internal medicine patients. We hypothesized that older internal medicine patients with an elevated level of NLR would be more likely to experience delirium.

## Methods

### Study design and population

We conducted this prospective cohort study at the Department of Geriatric (four floors), West China Hospital of Sichuan University from March of 2016 to July of 2017. Each floor is equipped with 65 beds and receives medical patients aged 60 years or older. Included patients were ≥ 70 years and had an anticipated length of stay of more than 2 days. Exclusion criteria were the presence of delirium on admission using the Confusion Assessment Method (CAM), inability to communicate due to severe deafness or severe dementia, a documented history of psychiatric illness, a terminal condition with life expectancy < 6 months, and incomplete data. The study was performed in accordance with the Declaration of Helsinki and approved by the Institutional Review Boards of West China Hospital, Sichuan University. Written informed consent was obtained from all participants. The present report complies with the Strengthening the Reporting of Observational Studies in Epidemiology (STROBE) statement, detailed in Additional file 1.

### Data collection

At baseline, all patients were assessed by trained research nurses within 24 h of admission. Demographic and general clinical characteristics including age, gender, living situation, education level, marriage status, smoking, alcohol intake, and type of admission were recorded. Peripheral blood samples were collected from patients to measure neutrophil and lymphocyte counts. The NLR was calculated by dividing the neutrophil count by the lymphocyte count. Severity of comorbidities was evaluated using the Charlson Comorbidity Index (CCI), a score based on 19 chronic diseases [23]. Patients were divided into mild (CCI 1–2), moderate (CCI 3–4) and severe (CCI ≥ 5) groups. Cognitive function was assessed using the SPMSQ (Short Portable Mental Status Questionnaire; score counts the number of errors, and all scores are adjusted by educational level: 0–2 errors indicate normal mental functioning; 3–4, mild cognitive impairment; 5–7, moderate cognitive impairment; and ≥ 8, severe cognitive impairment) [24]. Baseline functional status was measured by the Barthel Index for activities of living (ADL) [25]. Visual acuity and hearing ability were assessed with the Snellen eye chart and the whispered voice test, respectively.

### Outcome assessment

The outcome was the development of delirium during hospitalization. Research assessors were trained in screening delirium to ensure high rater reliability (kappa ≥ 0.9). Patients were screened for delirium by trained research assessors within 24 h of admission and every 48 h thereafter until discharge or for a maximum of 13 days. Delirium was assessed using the CAM based on the criteria of the *Diagnostic and Statistical Manual of Mental Disorders, Fourth Edition* (DSM-IV) [26, 27]. The CAM is a widely used diagnostic tool for delirium with a sensitivity of 94 %, a specificity of 89 %, and a Kappa’s inter-rater reliability between 0.70 and 1.00 [28]. The CAM is based on the following four features: (i) acute onset and fluctuating course; (ii) inattention; (iii) disorganized thinking; and (iv) altered level of consciousness. Patients were considered delirious if they displayed features (i) and (ii), with either (iii) or (iv). In case of doubt, an expert panel additionally screened patients according to the DSM-IV criteria to determine the final diagnosis.

### Statistical analysis

All data were entered and validated by two authors before the analysis. Descriptive data were expressed as number and percentage for categorical variables and as medians with the interquartile range (IQR) for continuous variables. Comparison between categorical variables was done using the chi-square test. Continuous variables were compared with the Mann-Whitney U-test or Kruskall-Wallis test. The predictive ability of NLR, neutrophil count, and lymphocyte count for delirium was assessed by the receiver operating characteristic (ROC) curve and compared using the Delong test [29]. The optimal cut-point value of each parameter for predicting delirium was determined based on the highest Youden index (sensitivity + specificity − 1). Participants were grouped based on NLR quartiles and the optimal cut-point value of NLR. Univariate and multivariate logistic regression analyses were performed to examine the association of NLR, neutrophil count, and lymphocyte count, all of which were modeled as categorical variables, with delirium. A multicollinearity diagnostic was conducted to assess the validity of the regression model by calculating the values of tolerance and the variance inflation factor (VIF). The tolerance > 0.1 and VIF < 10 were used to indicate no multicollinearity existed among the dependent variables. The multivariate logistic regression model was adjusted for age, sex, alcohol use, smoking, vision impairment, hearing impairment, cognitive impairment, disability, and CCI.

The median day of delirium occurrence in our study was three days after admission. We divided patients diagnosed with delirium into “early delirium” group if delirium occurred within three days, and “late delirium” group if delirium occurred more than three days after admission. Multinomial logistic regression analysis was further performed to test the associations between NLR and early delirium/late delirium.

Statistical analyses were performed using SPSS version 21.0 (IBM Crop., Armonk, NY) and MedCalc version 19.1 (MedCalc Software bv, Ostend, Belgium). *P*-value ≤ 0.05 was considered significant.

## Results

A total of 1,202 patients were recruited. We excluded patients who (1) had prevalent delirium (*n* = 33), (2) terminal condition (*n* = 107), (3) severe dementia (*n* = 113), (4) severe deafness (*n* = 119), (5) and missing data (*n* = 90). The final analytic sample consisted of 740 patients. Of these patients, the median age was 84 years (interquartile range 79–87 years) and the majority was male (71.2 %). The median NLR value and length of hospital stay were 3.1 (interquartile range 2.1–5.7) and 17 days (interquartile range 12–26 days), respectively. During hospital stay, 101 patients (13.6 %) were diagnosed with delirium. Other baseline characteristics of all patients are presented in Table 1.

**Table 1:** Baseline characteristics of the study participants

Characteristics	
**Demographic data**	
Age (years), median (IQR)	84 (79–87)
Male sex, n (%)	527 (71.2)
Living alone, n (%)	31 (4.2)
Married, n (%)	610 (82.4)
Education, n (%)	
Illiteracy or primary school	111 (15.0)
Middle school	133 (18.0)
High school or above	496 (67.0)
Alcohol abuse, n (%)	149 (20.1)
Smoker, n (%)	283 (38.2)
**Type of admission**	
Emergency admission, n (%)	97 (13.1)
**Primary admission diagnosis**	
COPD, n (%)	110 (14.9)
Hypertension, n (%)	83 (11.2)
Pneumonia, n (%)	79 (10.7)
Myocardial infarction, n (%)	63 (8.5)
Diabetes mellitus, n (%)	39 (5.3)
Stroke, n (%)	44 (5.9)
Heart failure, n (%)	22 (3.0)
Urinary tract infection, n (%)	15 (2.0)
Chronic renal failure, n (%)	12 (1.6)
Cataract, n (%)	10 (1.4)
Osteoporosis, n (%)	10 (1.4)
Peripheral vascular disease, n (%)	7 (0.9)
Atrial fibrillation, n (%)	6 (0.8)
Arthritis, n (%)	6 (0.8)
**Geriatric assessment**	
Vision impairment, n (%)	243 (32.8)
Hearing impairment, n (%)	229 (30.9)
Cognitive impairment, n (%)	243 (31.6)
Barthel index, median (IQR)	80 (55–95)
CCI, n (%)	
Mild (≤ 2)	566 (76.5)
Moderate (3–4)	136 (18.4)
Severe (≥ 5)	38 (5.1)

*IQR* interquartile range, *COPD* chronic obstructive pulmonary disease, *CCI* Charlson Comorbidity Index

**Fig. 1:**
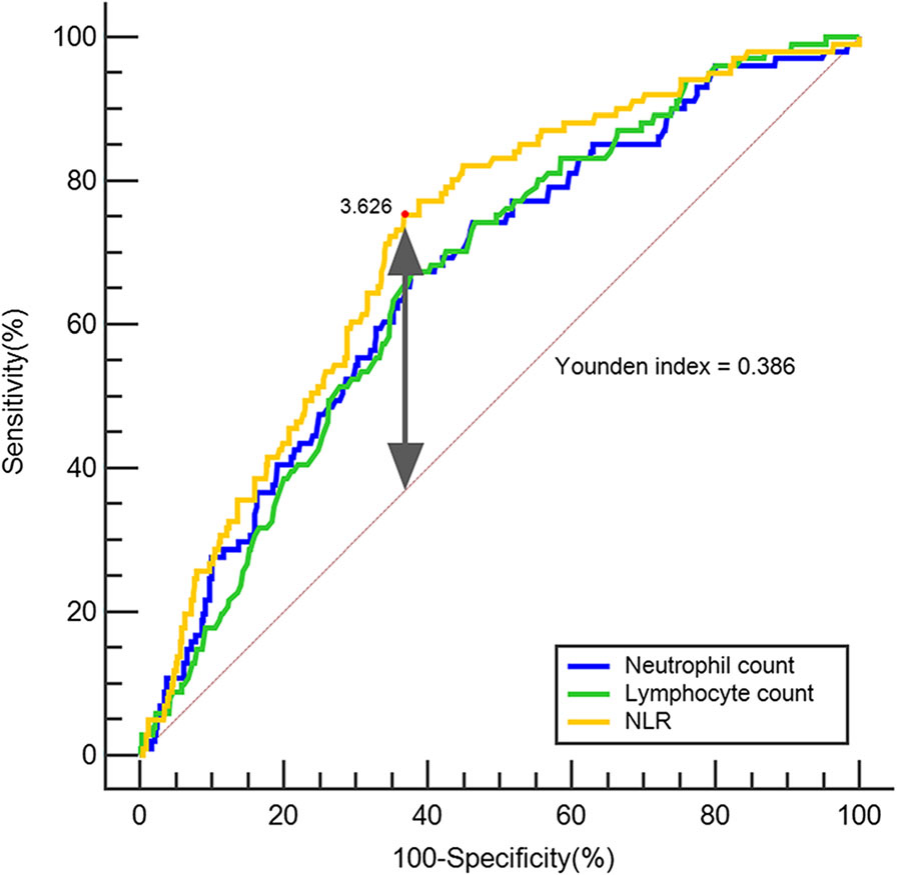
ROC curves for NLR (AUC (95 % confidence interval): 0.714 (0.66–0.77)), neutrophil count (0.667 (0.63–0.70)), and lymphocyte count (0.665 (0.63–0.70)), in predicting delirium. The best cut-point value of NLR to predict delirium was 3.626 (Youden index: 0.386). The curve of NLR demonstrated a larger AUC than the curves of neutrophil count and lymphocyte count

According to ROC curve analysis, the cut-point values were 3.626 for NLR (sensitivity, 75.2 %; specificity, 63.4 %*)*, 4.55 for neutrophil count (sensitivity, 67.3 %; specificity, 62.0 %), and 1.14 for lymphocyte count (sensitivity, 67.3 %; specificity, 62.3 %). Areas under the ROC curves (AUC) for NLR, neutrophil count, and lymphocyte count were 0.714 (*P* < 0.001), 0.667 (*P* < 0.001), and 0.665 (*P* < 0.001), respectively (Fig. 1). Based on the Delong test, NLR showed a significantly higher AUC than neutrophil count (Delong test, *P* = 0.047) and lymphocyte count (Delong test, *P* = 0.049).

Participants’ characteristics by the cut-point value of NLR are shown in Table 2. Compared to patients with NLR value ≤ 3.626, those with NLR value > 3.626 tended to be older, formally educated, smoking. Patients with NLR value > 3.626 had a higher emergency admission rate, higher rates of cognitive impairment, lower Barthel Index and higher CCI. In particular, significantly more patients with NLR > 3.626 were diagnosed with delirium than those with NLR ≤ 3.626.

**Table 2:** Patient characteristics by neutrophil-lymphocyte ratio (NLR) ≤ 3.626 and > 3.626

Characteristic	NLR ≤ 3.626 (*n* = 430)	NLR > 3.626 (*n* = 310)	*P*-value ^a^
Age (years), median (IQR)	83 (77–86)	85 (81–88)	< 0.001
Male sex, n (%)	297 (69.1)	230 (74.2)	0.129
Living alone, n (%)	17 (4.0)	14 (4.5)	0.706
Married, n (%)	364 (84.7)	246 (79.4)	0.062
Education, n (%)			
Illiteracy or primary school	58 (13.5)	53 (17.1)	
Middle school	69 (16.0)	64 (20.6)	0.022
High school or above	303 (70.5)	193 (62.3)	
Alcohol abuse, n (%)	84 (19.5)	65(21.0)	0.632
Smoker, n (%)	150 (34.9)	133 (42.9)	0.027
Vision impairment, n (%)	136 (31.6)	107 (34.5)	0.409
Hearing impairment, n (%)	127 (29.5)	102 (32.9)	0.328
Cognitive impairment, n (%)	89 (20.7)	145 (46.8)	< 0.001
Emergency admission, n (%)	25 (5.8)	72 (23.2)	< 0.001
Barthel index, median (IQR)	90 (70–100)	65 (40–85)	< 0.001
CCI, n (%)			
Mild (≤ 2)	344 (80.0)	222 (71.6)	
Moderate (3–4)	72 (16.7)	64 (20.6)	0.005
Severe (≥ 5)	14 (3.3)	24 (7.7)	
NLR, median (IQR)	2.2 (1.7–2.8)	6.6 (4.8–9.4)	< 0.001
Neutrophil (10^9^/L), median (IQR)	3.4 (2.6–4.2)	5.7 (4.6-8.0)	< 0.001
Lymphocyte (10^9^/L), median (IQR)	1.6 (1.2-2.0)	0.9 (0.6–1.1)	< 0.001
Delirium, n (%)	25(5.8)	76 (24.5)	< 0.001

*NLR* neutrophil-lymphocyte ratio, *CCI* Charlson Comorbidity Index, *IQR* interquartile range

Notes: ^a^ P values according to Mann-Whitney U or Chi-square tests

Participants’ characteristics by NLR quartiles are shown in Table 3. There were significantly differences in age, education, smoking, cognitive impairment, emergency admission, the Barthel Index and comorbidities among different NLR categories. Compared to those in the first quartile of NLR, patients in the 3rd and 4th quartiles of NLR had a greater proportion of delirium.

**Table 3:** Patient characteristics by quartile categorization

Characteristic	NLR Quartiles				*P*-value ^a^
	< 2.055 (*n* = 185)	2.055–3.110 (*n* = 185)	3.111–5.735 (*n* = 185)	> 5.735 (*n* = 185)	
Age (years), median (IQR)	83 (79–86)	83 (76–86)	85 (80–88)	85 (81–88)	< 0.001
Male sex, n (%)	126 (68.1)	125 (67.6)	144 (77.8)	132 (71.4)	0.110
Living alone, n (%)	8 (4.3)	6 (3.2)	10 (5.4)	7 (3.8)	0.758
Married, n (%)	160 (86.5)	155 (83.8)	153 (82.7)	142 (76.8)	0.091
Education, n (%)					
Illiteracy or primary school	17 (9.2)	31 (16.8)	27 (14.6)	36 (19.5)	
Middle school	24 (13.0)	31 (16.8)	39 (21.1)	39 (21.1)	0.002
High school or above	144 (77.8)	123 (66.5)	119 (64.3)	110 (59.5)	
Alcohol abuse, n (%)	149 (20.1)	84 (19.5)	65(21.0)	33 (17.8)	0.285
Smoker, n (%)	57 (30.8)	71 (38.4)	72 (38.9)	83 (44.9)	0.050
Vision impairment, n (%)	61 (33.0)	57 (30.8)	66 (35.7)	59 (31.9)	0.778
Hearing impairment, n (%)	53 (28.6)	58 (31.4)	58 (31.4)	60 (32.4)	0.879
Cognitive impairment, n (%)	33 (17.8)	41 (22.2)	68 (36.8)	92 (49.7)	< 0.001
Emergency admission, n (%)	5 (2.7)	14 (7.6)	27 (14.6)	51 (27.6)	< 0.001
Barthel index, median (IQR)	95 (75–100)	90 (65–100)	80 (47–95)	55 (35–80)	< 0.001
CCI, n (%)					
Mild (≤ 2)	152 (82.2)	151 (81.6)	141 (76.2)	122 (65.9)	
Moderate (3–4)	28 (15.1)	28 (15.1)	38 (20.5)	42 (22.7)	< 0.001
Severe (≥ 5)	5 (2.7)	6 (3.2)	6 (3.2)	21 (11.4)	
NLR, median (IQR)	1.6 (1.3–1.8)	2.6 (2.3–2.8)	4.0 (3.5–4.9)	8.3 (7.1–12.3)	< 0.001
Neutrophil count (10^9^/L), median (IQR)	2.9 (2.3–3.6)	3.7 (3.0-4.5)	4.7 (3.8–5.4)	6.6 (5.3-9.0)	< 0.001
Lymphocyte count (10^9^/L), median (IQR)	1.9 (1.5–2.4)	1.5 (1.2–1.8)	1.2 (0.9–1.4)	0.7 (0.6-1.0)	< 0.001
Delirium, n (%)	8 (4.3)	10 (5.4)	37 (20.0)	46 (24.9)	< 0.001

*NLR* neutrophil-lymphocyte ratio, *CCI* Charlson Comorbidity Index, *IQR* interquartile range

Notes: ^a^*P* values according Kruskall-Wallis or Chi-square tests

Univariate logistic regression showed a significant association of delirium with age, smoking, vision impairment, hearing impairment, cognitive impairment, Barthel Index, CCI, NLR, neutrophil count, and lymphocyte count (Table 4). In the multivariate regression model, NLR and lymphocyte count were independently associated with delirium. Additionally, when NLR was modeled as a categories variable based on quartiles, it was also found to be an independent predictor for delirium (Table 5). The tolerance and VIF were > 0.1 and < 10.0 for all dependent variables, respectively.

**Table 4:** Univariate logistic regression analysis of potential risk factors for delirium

Variable	Unadjusted OR (95 %CI)	*P*-value
Age (years)	1.14 (1.09–1.19)	< 0.001
Male sex	1.05(0.67–1.67)	0.826
Alcohol abuse	1.05 (0.62–1.76)	0.859
Smoker	1.71 (1.12–2.61)	0.013
Vision impairment	2.61 (1.71–3.99)	< 0.001
Hearing impairment	2.08 (1.36–3.20)	0.001
Cognitive impairment	56.95 (24.44-132.74)	< 0.001
Barthel index	0.94 (0.93–0.95)	< 0.001
CCI		
Mild (≤ 2)	Reference	
Moderate (3–4)	2.63 (1.61–4.30)	< 0.001
Severe (≥ 5)	6.76 (3.35–13.63)	< 0.001
Neutrophil count > 4.55 × 10^9^/L	3.70 (2.26–6.06)	< 0.001
Lymphocyte count ≤ 1.14 × 10^9^/L	3.36 (2.15–5.24)	< 0.001
NLR > 3.626	5.26 (3.26–8.50)	< 0.001
NLR quartiles		
<2.055	Reference	
2.055–3.110	1.26 (0.49–3.28)	0.630
3.111–5.735	5.53 (2.50-12.25)	< 0.001
> 5.735	7.32 (3.35–16.02)	< 0.001

*NLR* neutrophil-lymphocyte ratio, *CCI* Charlson Comorbidity Index, *OR* odds ratio, *CI* confidence interval

**Table 5:** Multivariate logistic regression analysis of potential risk factors for delirium

Variable	Adjusted OR (95 %CI) ^a^	*P*-value
Neutrophil count > 4.55 × 10^9^/L	1.30 (0.65–2.58)	0.455
Lymphocyte count ≤ 1.14 × 10^9^/L	3.10 (1.60-6.00)	0.001
NLR > 3.626	2.73 (1.40–5.34)	0.003
NLR quartiles		
<2.055	Reference	
2.055–3.110	0.61 (0.17–2.24)	0.457
3.111–5.735	3.75 (1.21–11.62)	0.022
> 5.735	2.18 (0.73–6.53)	0.165

*NLR* neutrophil-lymphocyte ratio, *OR* odds ratio, *CI* confidence interval

Notes: ^a^ Adjusted for age, sex, alcohol use, smoking, vision impairment, hearing impairment, cognitive impairment, disability, and CCI

Patients diagnosed with early delirium and those with late delirium had a similar median NLR (*P* = 0.188), but both groups had significantly higher NLR than those without delirium (early delirium vs. no delirium, *P* < 0.001; late delirium vs. no delirium, *P* = 0.001). NLR was independently associated with an increased risk of early delirium in the multinomial logistic regression analysis, but no significant relationship was noted between NLR and late delirium (Supplementary Table S1, Additional file 2)

## Discussion

This is the first prospective study to investigate the association between NLR, a marker for systemic inflammation, and delirium among older hospitalized patients. We found that the median level of NLR was elevated among elderly patients with delirium. Individuals with high NLR were more likely to experience delirium than those with low NLR. By showing that Increased NLR independently predicts greater risk of development of delirium. Taken together, these results suggest clinicians may be able to detect individuals who are at risk of adverse events and apply early intervention to prevent delirium.

Compared to geriatric medicine patients, post-surgical patients are at much greater risk of delirium (incidence: 11–51 %) [6]. We did not include older surgical patients because their NLR measures were relatively unstable due to surgery and anesthetic may have a impact on the inflammatory response. In previous studies, delirium was reported to be present in up to 20 % of older in-patients [2]. Our study found a relatively lower incidence of delirium (13.6 %). One plausible explanation could be that we only screened for delirium every 48 h, which might lead to omission of delirium cases.

Previous literature suggested that systemic inflammation and oxidative stress might be involved in the development of delirium [7, 8]. Previous studies have reported that inflammatory markers and cytokines can be detected in serum and cerebrospinal fluid among elderly patients with delirium [11, 30–32]. In addition, studies showed that inflammatory condition could negatively affect frontotemporal cognitive abilities such as memory, attention, and executive functions [33]. The initial immune response to stressful situations is characterized by systemic changes in leucocyte subtypes such as an increase in neutrophils and a decrease in lymphocytes [34], which can lead to an elevated level of NLR. In fact, the relationship of leucocyte subtypes with delirium has been investigated in previous studies. For example, the elevation of neutrophil count has been shown to be associated with delirium among older patients [35]. A cohort study found lower level of lymphocyte were to be an independent predictor of delirium [36]. Another recent study reported that patients with lower levels of lymphocyte were more likely to suffer from delirium [37].

NLR is emerging as a novel marker of systemic inflammation and it integrates information of both neutrophils and lymphocytes. NLR is different from traditional inflammatory markers (e.g., CRP, PCT, IL-6, IL-8, or TNF). NLR is a simple, inexpensive, and readily available inflammatory marker that can be directly derived from white blood cell (WBC) count on hospital admission. Furthermore, NLR is less likely to be influenced by fluid imbalance than the individual WBC subtypes [38]. Increased level of NLR has been identified as a more stronger predictor of adverse outcomes than traditional inflammation markers (e.g., CRP and WBC counts) in multiple studies [39–41]. In particular, NLR has been reported to have higher predictive value in delirium than CRP, neutrophils, and lymphocytes [42]. Therefore, NLR might be better at reflecting a. association between systemic inflammation and delirium than neutrophil or lymphocyte alone.

Few studies have investigated the association between NLR and delirium. Egberts et al. [42] found that an elevated level of NLR was an independent predictor of delirium among acutely ill elderly patients. Kotfits et al. [43] revealed that an increased level of NLR is significantly associated with increased risk of delirium in patients with acute ischemic stroke. The results of our study are consistent with these previous findings. Moreover, there are studies evaluating the role of NLR as a risk factor for cognitive impairment. Halazun et al. [44] demonstrated a contribution of NLR to cognitive dysfunction after carotid endarterectomy. The relationship of NLR with cognitive impairment has been suggested by Liu et al. and higher NLR was also found in patients with cognitive decline [45]. Our results added further evidence to the relationship of NLR with delirium or cognitive impairment.

Interestingly, we found NLR to be an independent predictor of early but not late delirium. One plausible explanation is that anti-inflammatory treatment, severity of illness, and stress situation may lead to the dynamic change of NLR, and assessing NLR on admission may only reflect the current inflammatory status. These results supported previous study that found increased NLR was a potential marker for prediction of early-onset delirium in patients with acute ischemic stroke [43].

The mechanisms of how systemic inflammation results in delirium are still unclear. Inflammation characterized by increased neutrophils and decreased lymphocytes can reduce plaque stability and promote atherosclerosis, which may increase the risk of delirium through microinfarcts. Furthermore, reactive oxygen species released by neutrophils lead to disruption of the blood-brain barrier (BBB) and increase its permeability, cytokines then migrate across the BBB and activate microglia which will produce reactive oxygen species, the accumulation of cytokines and reactive oxygen species in brain may lead to the process of oxidization and inflammation and eventually result in neurodegeneration [7, 9].

This study was conducted with a large sample of older hospitalized patients, which may reduce selection bias. However, our study had several limitations. First, this was a single-center study that might be insufficient to represent a general population of older patients with delirium. Second, a single measurement of NLR on admission does not allow for evaluating the stability of this marker over time and assessing the long-term effect of this marker on delirium. Third, other inflammatory markers (e.g., CRP, IL-6, or IL-8), which may have influence on delirium, were not included in our study.

## Conclusions

In this study, we found that elevated NLR was significantly associated with increased odds of delirium in older internal medicine patients. The results suggest that NLR can serve as a convenient, inexpensive, and rapidly accessible marker to predict delirium. The findings of this study also underlines that systemic inflammation and oxidative stress play a key role in the pathophysiology of delirium. Thus, use of this marker in routine clinical research can help clinicians identify patients who at risk of delirium, and may help to prevent negative outcomes.

## Supplementary information

**Additional file 1.**

**Additional file 2.**

## Data Availability

The datasets used for the current study are available from the corresponding author upon reasonable request.

## Abbreviations

NLR: Neutrophil-lymphocyte ratio
SPMSQ: Short Portable Mental Status Questionnaire
CCI: Charlson Comorbidity Index
CAM: Confusion Assessment Method
CRP: C-reactive protein
TNF: Tumor necrosis factor
