# Supplementary material for: Neutrophil-lymphocyte ratio as a predictor of delirium in older internal medicine patients: a prospective cohort study
Source: BMC Geriatr. 2021 May 25;21:334. doi: 10.1186/s12877-021-02284-w (PMC8147036; doi:10.1186/s12877-021-02284-w)
Supplement: Supplementary file 2 — Additional file 2. [file 12877_2021_2284_MOESM2_ESM.docx]

**Supplementary Table S1**. Multinomial logistic regression analysis of NLR associated with early delirium/late delirium

|  | Early delirium | | Late delirium | |
| --- | --- | --- | --- | --- |
| Variable | Adjusted OR (95%CI) ^a^ | *P*-value | Adjusted OR (95%CI) ^a^ | *P*-value |
| NLR >3.626 | 6.38 (2.43-16.78) | <0.001 | 1.47 (0.67-3.22) | 0.333 |
| NLR quartiles |  |  |  |  |
| <2.055 | Reference |  | Reference |  |
| 2.055-3.110 | 0.79 (0.10-6.13) | 0.821 | 0.62 (0.14-2.70) | 0.525 |
| 3.111-5.735 | 8.97 (1.49-54.07) | 0.017 | 2.40 (0.68-8.48) | 0.175 |
| >5.735 | 5.62 (0.96-32.78) | 0.055 | 1.20 (0.35-4.20) | 0.771 |

*NLR* neutrophil-lymphocyte ratio, *OR* odds ratio, *CI* confidence interval.

Notes: ^a^ Adjusted for age, sex, alcohol use, smoking, vision impairment, hearing impairment, cognitive impairment, disability, and CCI.
